# Evaluation of the competitiveness of the container multimodal port hub

**DOI:** 10.1038/s41598-022-23845-y

**Published:** 2022-11-11

**Authors:** Min Wan, Haibo Kuang, Yue Yu, Ruirui Zhang

**Affiliations:** 1 School of Maritime Economics and Management, Dalian Maritime University, Dalian, 116000 China; 2 Collaborative Innovation Center for Transport Studies, Dalian Maritime University, Dalian, 116000 China

**Keywords:** Engineering, Mathematics and computing

## Abstract

The research on the competitiveness of container multimodal transport port hub, comprehensively considering the software and hardware strength of port hub, is of great significance to analyze the specific source competitiveness of port hub, the optimal allocation of multimodal transport capacity resources and port operation management planning. Based on this, according to the principle of fuzzy set and membership function in fuzzy theory, this paper adopts the modeling method of multi-attribute decision-making to deeply analyze the key influencing factors of port hub competitiveness, extract the core indicators from the four aspects of business capacity, transport capacity resources, infrastructure and service quality, and then construct the fuzzy multi-attribute decision-making evaluation model of port hub competitiveness, and take the container transportation in Northeast China as an example. The research shows that the key influencing factors of multimodal transport port hub competitiveness have changed from traditional infrastructure factors to flexible influencing factors such as transportation service quality, transportation capacity and resource integration ability, transportation business efficiency, and port enterprises are in the critical period of industry transformation and upgrading. Strengthening inter industry integration, building industrial ecological alliance and building a multi-mode transportation integrated service chain with port as the core play an obvious role in promoting the formation of the core competitiveness of port hub. Among the ports along the coast of Liaoning, Yingkou Port has the strongest competitiveness against the supply of corn containers in Northeast China, and the competitiveness of other ports from strong to weak is Jinzhou port, Beiliang port, Panjin Port, Huludao port, Dandong port, Dalian port and Suizhong port. The research results enrich the theoretical system of multimodal transport port hub competitiveness research, and provide reference suggestions for port enterprises, transportation enterprises, government departments and other relevant subjects in strategy formulation, policy implementation, layout planning and so on.

## Introduction

The traditional research on port competitiveness focuses on the analysis of port competitiveness alone and lacks the research on port hub competitiveness from the perspective of multimodal transport network and industrial relevance. In view of the current construction demand of China’s comprehensive transportation system and the development trend of multimodal transport, it is urgent to break through the traditional research perspective and method of port competitiveness and study the competitiveness of port hubs in multimodal transport network. Therefore, it is necessary to build the competitiveness evaluation model of container multimodal transport port hub based on the analysis of the characteristics of regional port hub and the development needs of container multimodal transport network, to provide strong support for the differentiated development between ports and the optimization of multimodal transport network.

The research on the competitiveness evaluation of multimodal transport hubs is extensive, which can be summarized into three aspects: the perspective analysis of hub competitiveness, the analysis of influencing factors and the construction of competitiveness evaluation model. Firstly, from the perspective of port hub competitiveness research, Li et al.^1–6^ carried out research on logistics hub competitiveness at the world level, national level, multimodal transport industry level and port enterprise level, respectively. Forte et al.^7–10^ conduct competitiveness research on the dimensions of cost control and economic feasibility for specific multimodal transport markets such as roro market and short-distance marine transport market. Han et al.^11,12^ studied port competitiveness from the perspective of optimizing the enterprise supply chain. Wiegmans et al.^13^ studied the competitiveness of inland multimodal transport hubs from the perspective of cargo loading equipment volume ratio and container unloading and transfer efficiency. Second, in terms of research on influencing factors of port competitiveness, Wang et al.^14–16^ analyzed the driving factors of port competitiveness from the perspective of multi-flow convergence of logistics, people flow, business flow, capital flow and information flow based on the interactive relationship between port and city. Da Cruz et al.^17–19^ studied the effects of port infrastructure construction, port operation efficiency and hinterland accessibility construction on the improvement of port competitiveness from the perspective of port managers. Sun et al.^20^ studied mechanism of different price guidance mechanisms on improving the competitiveness of multimodal transport schemes. Santos et al.^21–23^ studied the effects of subsidies, costs, site selection and other factors on the competitiveness of multimodal transport hubs. Mao^24,25^ studied the competitiveness of multimodal transport services from the perspective of transport efficiency and externality. Third, the construction of competitiveness evaluation model. Lagoudis et al.^26,27^ reviewed and analyzed the existing literature on port selection, port productivity and port competitiveness, and found that the existing research models can be divided into three types: qualitative analysis, quantitative analysis and combination of quantitative and qualitative analysis. For example, Zhang^28^ used discrete variation TOPSIS matter-element method to study port competitiveness. Liu et al.^29^ used AHP-FCE-DEA model to study the competitiveness of port enterprises and logistics enterprises in multimodal transport. Zhang et al.^30^ constructed a mathematical model to study the competitiveness of multimodal transport hub from the perspective of hub comprehensive service level and generalized service cost equilibrium. Kong et al.^31^ studied port competitiveness and sustainable development capacity by using relaxation measurement model from the perspective of port interaction. At the same time, in the research of multimodal transport and comprehensive transport trends, the collaborative operation of multiple modes of transport, logistics intelligence, and the integration and analysis of transportation big data have become the frontiers of the industry development, reflecting the importance and practical needs of analyzing the competitiveness of port hubs based on the perspective of multimodal transport system^32–35^.

According to the analysis of the existing documents, compared with the traditional port competitiveness research, the research on the competitiveness of container multimodal transport port hub mainly includes the following three difficulties: first, unlike the traditional port competitiveness which focuses on the throughput capacity of the port itself, the competitiveness of multimodal transport port hub is closely related to the efficient integration ability of various modes of transportation, Therefore, how to make a comprehensive and objective analysis of the regional comprehensive transportation system with the port as the hub is one of the difficulties in the research. Second, compared with a single port, the multimodal transport port hub involves a variety of stakeholders, and the complex competition and cooperation relationship among the stakeholders jointly determines the competitiveness level of the port hub. Therefore, how to build a scientific and reasonable evaluation index system and deeply analyze the influence and role of the multi-agent of the multimodal transport port hub is the second difficulty in the research. Third, unlike the traditional port competitiveness, which is mostly quantitative evaluation model, the multimodal port hub competitiveness is reflected in the comprehensive strength of service quality soft power and transport capacity resources hard power. Therefore, how to build a mathematical model that can comprehensively analyze the comprehensive strength of port hub hardware and software becomes the key of this study.

The hesitant fuzzy multi-attribute decision-making method is based on the theory of interval number separation degree and interval number comparison possibility degree. By constructing a hesitant fuzzy set, it can effectively deal with the multi-value problem of single element membership degree, and has obvious advantages in dealing with the integration of quantitative data and interval data, it can build an evaluation model that effectively reflects the soft and hard strength of the port. Moreover, multi-attribute decision-making method is widely used in marine cost accounting, stock investment decision-making, green supplier selection and other related fields, with high adaptability and feasibility^36–39^. Therefore, this paper uses the hesitant fuzzy multi-attribute decision-making method to deeply analyze the influencing factors of port hub competitiveness and construct the evaluation index system of port hub competitiveness around the four aspects of business capacity, transportation capacity resources, infrastructure and service quality. In view of the pattern of “grain transportation from the north to the South” in China’s grain transportation, and in combination with the development needs of the multimodal transport system in Northeast China^40,41^, this paper takes the coastal container multimodal transport port hub in Northeast China as the research object, and takes the transportation of major grain products in Northeast China as the realistic scenario, and conducts an empirical analysis on the competitiveness of corn container transportation in the multimodal transport port hub.

This paper will follow the following steps. First, build the competitiveness evaluation model of port hub; secondly, design the solution method of port hub competitiveness evaluation model; thirdly, taking the corn container transportation in Northeast China as an example, this paper makes an empirical analysis; fourth, analyze the empirical results and get the research conclusions.

## Model construction

### Concept definition

The competitiveness evaluation of multimodal transport port hub belongs to multiattribute decision-making problem. The competitiveness evaluation index system includes quantitative indices and qualitative indices. Therefore, two concepts of interval number separation degree and interval number comparison possibility degree need to be introduced for competitiveness evaluation^42^.

Definition 1: Set interval numbers $a = [a^{l} ,a^{u} ]$,$b = [b^{l} ,b^{u} ]$, and regard $D(a,b) = \left\| {a - b} \right\| = \left| {b^{l} - a^{l} } \right| + \left| {b^{u} - a^{u} } \right|$ as the distance degree of interval number a, b.

Example 1: The weight vector $W$ solution follows the idea of maximizing the overall deviation value of the evaluation object under the evaluation index set. Under a certain index $u_{j} \in U$, the deviation value of evaluation object $x_{i}$ and other evaluation objects are expressed by $L_{ij} (w)$, while $L_{i} (w)$ represents the sum of deviation values of all evaluation objects. Further build the overall deviation function of evaluation object under all indexes.

1 $$L_{ij} (w)\,{ = }\,\sum\limits_{{{\text{k}} = 1}}^{n} {\left( {\left| {r_{ij}^{l} - r_{kj}^{l} } \right| + \left| {r_{ij}^{u} - r_{kj}^{u} } \right|} \right)} w_{j} , \, i \in N{,}\, \, j \in M$$

2 $$L_{i} (w)\,{ = }\,\sum\limits_{i = 1}^{n} {L_{ij} (w)\, = \,} \sum\limits_{i = 1}^{n} {\sum\limits_{{{\text{k}} = 1}}^{n} {\left( {\left| {r_{ij}^{l} - r_{kj}^{l} } \right| + \left| {r_{ij}^{u} - r_{kj}^{u} } \right|} \right)} w_{j} } , \, i \in N$$

3 $$L(w)\,{ = }\,\sum\limits_{j = 1}^{m} {L_{i} (w)\, = } \,\sum\limits_{j = 1}^{m} {\sum\limits_{i = 1}^{n} {\sum\limits_{{{\text{k}} = 1}}^{n} {\left( {\left| {r_{ij}^{l} - r_{kj}^{l} } \right| + \left| {r_{ij}^{u} - r_{kj}^{u} } \right|} \right)} w_{j} } }$$

Definition 2: let $a = [a^{l} ,a^{u} ]$,$b = [b^{l} ,b^{u} ]$, and $S(a) = a^{u} - a^{l}$, $S(b) = b^{u} - b^{l}$.

4 $$P(a \ge b) = \frac{{\min \left\{ {S(a) + S(b)} \right.,\left. {\max (a^{u} - b^{l} ,0)} \right\}}}{S(a) + S(b)}$$

Define the possibility that $P(a \ge b)$ is $a \ge b$. In this definition, $P(a \ge b)$ has the following properties:

- (1)$$P(a \ge b) + P(b \ge a) = 1$$
- (2) If $$P(a \ge b) = P(b \ge a)$$, then $$P(a \ge b) = P(b \ge a) = 1/2$$.
- (3) If $a^{U} \le b^{L}$, then $P(a \ge b) = 0$; If $a^{L} \ge b^{U}$, then $P(a \ge b) = 1$.
- (4) For three interval numbers $a,b,c$, if $a \ge b$, then $P(a \ge c) \ge P(b \ge c)$.

### Index selection

The competitiveness evaluation index system of multimodal transport hub needs to focus on the development level of the integration capacity of various modes of transportation, the port handling and transfer capacity, and the service efficiency of multimodal transport. On the basis of fully referring to the relevant research literature on port hub competitiveness evaluation^43–45^, this paper constructs a container multimodal transport port hub competitiveness evaluation index system including 12 three-level indicators from four aspects of business capacity, transport capacity resources, infrastructure and service quality. Among them, the business capacity focuses on the proportion of containerized transport in the port, the proportion of sea rail transport in the container and the price of multimodal transport in the container. The transport capacity resources mainly focus on the transport capacity of three different modes of transport in the multimodal transport network: Road, railway and water transport. The infrastructure focuses on the storage capacity in the transport network and the port’s handling and transfer capacity. As shown in Table 1.

**Table 1:** Competitiveness evaluation index system of container multimodal transport port hub.

First level indicators	Secondary index	Three level indicators	type
Competitiveness of container multimodal transport port hub	Business capability	Sea rail intermodal container volume/TEU (X1)	Benefit type quantitative index
		Multimodal transport price/yuan (X2)	Cost quantitative index
		Containerization rate (X3)	Benefit type quantitative index
	Capacity resources	Container truck (X4)	Benefit type quantitative index
		Railway transportation distance /km (X5)	Cost quantitative index
		Liner route (X6)	Benefit type quantitative index
	Infrastructure resources	Storage capacity/10,000 tons (X7)	Benefit type quantitative index
		Sea rail intermodal container handling capacity/TEU (X8)	Benefit type quantitative index
		Port berth handling capacity/TEU (X9)	Benefit type quantitative index
	Service quality	User satisfaction (X10)	Benefit type qualitative index
		Policy guidance (X11)	Benefit type qualitative index
		Enterprise strategic planning (X12)	Benefit type qualitative index

Service quality is a secondary evaluation index that describes the competitive soft power of the container multimodal transport port hub, including three tertiary indicators: user satisfaction, policy guidance and enterprise strategic planning. Among them, user satisfaction refers to the overall evaluation of the multimodal transport system with the port hub as the core by the cargo owners, ship owners, logistics enterprises and other subjects. The policy guidance strength refers to the support of the governments at all levels to the port hub in terms of policy dividends and development positioning. The enterprise strategic planning strength refers to the specific layout of the port function positioning within the port group. For the above three interval indicators, this paper uses the scoring method of 1 to 10 points to measure. Through the questionnaire survey, the government, ship owners, port groups, cargo owners, logistics enterprises and other stakeholders are investigated, so as to obtain the data required for this study. With reference to the scoring method of Likert scale, the scores from 1 to 10 are divided into the following nine sections. The corresponding descriptions of the standards of different sections are shown in Table 2.

**Table 2:** Fuzzy interval division standard and description.

Interval standard	Description of qualitative index interval standard		
	User satisfaction	Policy guidance	Enterprise strategic planning
[1, 2]	Terrible	Terrible	Terrible
[2, 3]	Very poor	Very poor	Very poor
[3, 4]	Quite poor	Quite poor	Quite poor
[4, 5]	Poor	Poor	Poor
[5, 6]	General	General	General
[6, 7]	Good	Good	Good
[7, 8]	Quite good	Quite good	Quite good
[8, 9]	Very good	Very good	Very good
[9, 10]	Great	Great	Great

### Solution steps

#### Building decision matrix

Set $X = \left\{ {x_{1} ,x_{2} \cdot \cdot \cdot ,x_{n} } \right\}$ as the evaluation object set, $U = \left\{ {u_{1} ,u_{2} \cdot \cdot \cdot ,u_{m} } \right\}$ as the evaluation index set, $W = \left\{ {w_{1} ,w_{2} \cdot \cdot \cdot ,w_{m} } \right\}^{T}$ as the weight vector of evaluation index,${\text{w}}_{i} \ge 0$, ${\text{w}}_{i} \in [w_{i}^{l} ,w_{i}^{u} ]$, $\sum\nolimits_{i = 1}^{m} {w_{i} } \, = \,1$. This paper uses evaluation index $u_{j} \in U$ to evaluate evaluation object $x_{i} \in X$, get the accurate evaluation value $m_{1}$ and interval evaluation value $m_{2}$($m_{1} + m_{2} = m$). Furthermore, the decision matrix $A\, = \,(a_{ij} )_{n \times m} \, = \,[(a_{ij} )_{{n \times m_{1} }} ,\,(a_{ij}^{l} ,\,a_{ij}^{u} )_{{n \times m_{2} }} ]$ can be obtained by applying all the evaluation indexes to evaluate all the evaluation objects.

#### Normalized decision matrix

As mentioned above, indicators can be divided into accurate type (Quantitative) and interval type (Qualitative) according to the type of indicator data value. At the same time, indicators can be divided into cost type and benefit type according to the change of indicator value and the changing trend of evaluation results under the indicator. Therefore, indicator types can be divided into four types: quantitative cost type, quantitative benefit type, qualitative cost type and qualitative benefit type. On the basis of the references^42,46^, different types of data are standardized as follows.

Standardization of quantitative index data: formulas (5) and (6) are cost-effective and benefit oriented index data standardization formulas respectively.

5 $$r_{ij} = \frac{{\mathop {\min }\limits_{j} \, a_{ij} }}{{{\text{a}}_{ij} }} \, j \in N,N = \left\{ {1,2,...,n} \right\}$$

6 $$r_{ij} = \frac{{a_{ij} }}{{\mathop {\max }\limits_{j} {\text{ a}}_{ij} }} \, j \in N,N = \left\{ {1,2,...,n} \right\}$$

Standardization of qualitative index data: formulas (7) and (8) are cost-effective and benefit oriented index data standardization formulas respectively.

7 $$\left\{ \begin{gathered} r_{ij}^{l} = (1/a_{ij}^{u} )/\sqrt {\sum\limits_{j = 1}^{n} {(1/a_{ij}^{l} )^{2} } } \hfill \\ r_{ij}^{u} = (1/a_{ij}^{l} )/\sqrt {\sum\limits_{j = 1}^{n} {(1/a_{ij}^{u} )^{2} } } \hfill \\ \end{gathered} \right. \, j \in N,\,N\, = \,\left\{ {1,2,...,n} \right\}$$

8 $$\left\{ \begin{gathered} {\text{r}}_{ij}^{l} = a_{ij}^{l} /\sqrt {\sum\limits_{j = 1}^{n} {(a_{ij}^{u} )^{2} } } \hfill \\ r_{ij}^{u} = a_{ij}^{u} /\sqrt {\sum\limits_{j = 1}^{n} {(a_{ij}^{l} )^{2} } } \hfill \\ \end{gathered} \right. \, j \in {\text{N, N = }}\left\{ {1,2,...,n} \right\}$$

Based on formula (5), (6), (7) and (8), decision matrix $A = (a_{ij} )_{n \times m} = [(a_{ij} )_{{n \times m_{1} }} ,(a_{ij}^{l} ,a_{ij}^{u} )_{{{\text{n}} \times m_{2} }} ]$ can be transformed into normalized matrix $R = (r_{ij} )_{n \times m} = [(r_{ij} )_{{n \times m_{1} }} ,(r_{ij}^{l} ,r_{ij}^{u} )_{{n \times m_{2} }} ]$.

#### Solve weight vector

According to formulas (1), (2) and (3), solving the weight vector $W$ is to solve the maximum $L(w)$ value problem under certain constraints. The solution equation is as follows.

9 $$\begin{gathered} \max L(w)\, = \,\sum\limits_{j = 1}^{{\text{m}}} {\sum\limits_{i = 1}^{n} {\sum\limits_{k = 1}^{n} {\left( {\left| {r_{ij}^{l} - r_{kj}^{l} } \right| + \left| {r_{ij}^{u} - r_{kj}^{u} } \right|} \right)} } } w_{i} \hfill \\ s.t.{\text{ w}}\,{ = }\,{\text{(w}}_{1} {\text{,w}}_{2} {,} \cdot \cdot \cdot {\text{,w}}_{m} {)}^{T} \hfill \\ w_{j} \in [w_{j}^{l} ,w_{j}^{u} ],w_{j} \ge 0,\sum\limits_{j = 1}^{m} {w_{j} \, = \,1} \hfill \\ \end{gathered}$$

#### Solve the comprehensive index value

The comprehensive index value ${\text{z}}_{i}$ is the sum of the index evaluation values of each port in the competitiveness index evaluation system of multimodal transport hub, and the calculation formula is as follows.

10 $$z_{{\text{i}}} = \sum\limits_{j = 1}^{m} {r_{ij} w_{j} ,j \in M}$$

where $w_{j}$ is the weight of the jth index,${\text{r}}_{ij}$ is the evaluation value in the normalized matrix $R = (r_{ij} )_{n \times m} = [(r_{ij} )_{{n \times m_{1} }} ,(r_{ij}^{L} ,r_{ij}^{U} )_{{n \times m_{2} }} ]$.

#### Constructing possibility complementary matrix

Through formula (9), the comprehensive index of each port is interval data, and further use formula (4) to obtain the possibility complementary judgment matrix $P\, = \,(P_{ij} )n\, \times \,n$.

#### Solve sorting vector

On the basis of obtaining the possibility degree complementary judgment matrix of each port, the ranking vector of the possibility degree matrix P is obtained by using the solution formula of the ranking vector $h = (h_{1} ,h_{2} , \cdot \cdot \cdot ,h_{n} )^{T}$ of the fuzzy complementary judgment matrix, and the competitiveness level of coastal ports in Northeast China is ranked according to the component size of the ranking vector.

11 $$h_{i} = \frac{{\left( {\sum\limits_{j = 1}^{n} {P_{ij} + \frac{n}{2} - 1} } \right)}}{n(n - 1)}$$

In the evaluation of the competitiveness of port hubs in the multimodal transport network, we should consider not only the quantitative indicators such as container sea rail transport volume, multimodal transport price, containerization rate, highway cargo collection capacity, railway transport distance and liner routes, but also the qualitative indicators reflecting the soft power of port competition such as user satisfaction, policy guidance and enterprise strategic planning, It is necessary to analyze the mixed data composed of accurate data and interval data. Therefore, according to the principle of maximizing the interval separation degree and scheme attribute deviation, the weight vector and comprehensive index value are solved by constructing the decision matrix, and the ranking vector is further solved to rank the competitiveness of each port.

## Empirical analysis

Taking eight coastal ports in Northeast China as the research object, taking the northeast corn container multimodal transport as the application scenario, and using the hesitation fuzzy multi-attribute decision-making method, this paper constructs the competitiveness evaluation model of each port in attracting corn container supply. According to the evaluation index system shown in Table 1 and the fuzzy interval division standard shown in Table 2, the decision matrix shown in Table 3 is obtained by using the decision matrix construction method.

**Table 3:** Decision matrix.

	Dalian port A	Yingkou port B	Jinzhou port C	Beiliang port D	Dandong port E	Huludao port F	Panjin port G	Suizhong port H
X1	230	2570	2300	450	390	622	605	148
X2	203	200.2	201.2	199.3	205.2	202.3	203.6	202.9
X3	1.23%	56.39%	42.48%	1.79%	1.13%	6.94%	25%	13.50%
X4	1700	1770	3200	3400	500	64	50	15
X5	876.50	683.30	750.30	853.10	726.20	776.30	681.60	869.70
X6	2	4	2	2	2	4	4	2
X7	82.5	470.5	252	197	150	41.2	138.8	28
X8	10	24	8	24	24	50	40	50
X9	18	13	3	25	21	22	26	12
X10	[6, 7]	[8, 9]	[8, 9]	[7, 8]	[6, 7]	[5, 6]	[5, 6]	[4, 5]
X11	[4, 5]	[9, 10]	[8, 9]	[8, 9]	[5, 6]	[5, 6]	[4, 5]	[4, 5]
X12	[4, 5]	[8, 9]	[7, 8]	[7, 8]	[5, 6]	[5, 6]	[5, 6]	[4, 5]

On the basis of constructing the decision matrix, for the benefit type quantitative index, cost type quantitative index and benefit type qualitative index, the formulas (5), (6), (7) and (8) are respectively applied for standardization to obtain the standardized decision matrix as shown in Table 4.

**Table 4:** Standardized decision matrix.

	Dalian port A	Yingkou port B	Jinzhou port C	Beiliang port D	Dandong port E	Huludao port F	Panjin port G	Suizhong port H
X1	0.09	1.00	0.89	0.18	0.15	0.24	0.24	0.06
X2	0.98	1.00	0.99	1.00	0.97	0.99	0.98	0.98
X3	0.02	1.00	0.75	0.03	0.02	0.12	0.44	0.24
X4	0.50	0.52	0.94	1.00	0.15	0.02	0.01	0.00
X5	0.78	1.00	0.91	0.80	0.94	0.88	1.00	0.78
X6	0.50	1.00	0.50	0.50	0.50	1.00	1.00	0.50
X7	0.18	1.00	0.54	0.42	0.32	0.09	0.30	0.06
X8	0.20	0.48	0.16	0.48	0.48	1.00	0.80	1.00
X9	0.69	0.50	0.12	0.96	0.81	0.85	1.00	0.46
X10	[0.29,0.39]	[0.39,0.51]	[0.39,0.51]	[0.34,0.45]	[0.29,0.39]	[0.24,0.34]	[0.24,0.34]	[0.19,0.28]
X11	[0.19,0.28]	[0.44,0.56]	[0.39,0.51]	[0.39,0.51]	[0.24,0.34]	[0.24,0.34]	[0.19,0.28]	[0.19,0.28]
X12	[0.19,0.28]	[0.39,0.51]	[0.34,0.45]	[0.34,0.45]	[0.24,0.34]	[0.24,0.34]	[0.24,0.34]	[0.19,0.28]

According to definition 1, the index weight in the port competitiveness evaluation model is solved by using the concept of interval distance, and the single objective optimization model as shown below is constructed according to formulas (1), (2), (3) and (9).

$${\text{MaxD}}\left( {\text{w}} \right)\, = \,{1}0.{\text{96w1}}\, + \,{\text{14w2}}\, + \,{11}.{\text{6w3}}\, + \,{11}.{\text{26w4}}\, + \,{13}.{\text{37w5}}\, + \,{13}.{\text{63w6}}\, + \,{1}0.{\text{97w7}}\, + \,{13}.{\text{43w8}}\, + \,{12}.{\text{4w9}}\, + \,{5}.{\text{24w1}}0\, + \,{6}.{\text{29w11}}\, + \,{5}.{\text{64w12}}{.}$$

$${\text{s}}.{\text{t}}.0.0{8}\, \le \,{\text{w1}}\, \le \,0.{16},0.0{6}\, \le \,{\text{w2}}\, \le \,0.{14},0.0{7}\, \le \,{\text{w3}}\, \le \,0.{15},0.{1}0\, \le \,{\text{w4}}\, \le \,0.{16},0.0{8}\, \le \,{\text{w5}}\, \le \,0.{14},0.0{6}\, \le \,{\text{w6}}\, \le \,0.{14},0.0{8}\, \le \,{\text{w7}}\, \le \,0.{16},0.0{8}\, \le \,{\text{w8}}\, \le \,0.{14},0.0{8}\, \le \,{\text{w9}}\, \le \,0.{12},0.0{7}\, \le \,{\text{w1}}0\, \le \,0.{12},0.0{8}\, \le \,{\text{w11}}\, \le \,0.{16},0.0{6}\, \le \,{\text{w12}}\, \le \,0.{14}$$

$$\sum\limits_{i = 1}^{12} {w_{i} \, = \,1,} \, w_{i} \ge 0, \, i \in \,(1,2,3,...12)$$

For the single objective maximization optimization model of port competitiveness, the model is solved by using Python 2.7 software, and the optimal weight vector is obtained as follows.

$${\text{w}}\, = \,(0.0{8},0.{14},0.0{7},0.{1},0.0{8},0.0{8},0.0{8}, \, 0.0{8}, \, 0.0{8}, \, 0.0{7}, \, 0.0{8}, \, 0.0{6}).$$

According to formula (10), the comprehensive index value $z_{j} (w)(j \in N)$ is

$$z_{{\text{i}}} = \sum\limits_{j = 1}^{m} {r_{ij} w_{j} ,j \in M}$$

$${\text{z}}_{{\text{A}}} \left( {\text{w}} \right)\, = \,\left[ {0.{4315},0.{45}0{8}} \right],{\text{ z}}_{{\text{B}}} \left( {\text{w}} \right)\, = \,\left[ {0.{7454},0.{77}0{6}} \right],{\text{ z}}_{{\text{C}}} \left( {\text{w}} \right)\, = \,\left[ {0.{6136},0.{6378}} \right],{\text{ z}}_{{\text{D}}} \left( {\text{w}} \right)\, = \,\left[ {0.{5845},0.{6}0{81}} \right],{\text{ z}}_{{\text{E}}} \left( {\text{w}} \right)\, = \,\left[ {0.{4624},0.{4828}} \right],{\text{ z}}_{{\text{F}}} \left( {\text{w}} \right)\, = \,\left[ {0.{5239},0.{5437}} \right],{\text{ z}}_{{\text{G}}} \left( {\text{w}} \right)\, = \,\left[ {0.{5633},0.{5825}} \right],{\text{ z}}_{{\text{H}}} \left( {\text{w}} \right)\, = \,\left[ {0.{4246},0.{4429}} \right],$$

According to formula (4), calculate the possibility of comparing the comprehensive index values of port competitiveness, and establish the possibility matrix.

$$p = \left\{ {\begin{array}{*{20}c} {0.5} & 0 & 0 & 0 & 0 & 0 & 0 & {0.6975} \\ 1 & {0.5} & 1 & 1 & 1 & 1 & 1 & 1 \\ 1 & 0 & {0.5} & 1 & 1 & 1 & 1 & 1 \\ 1 & 0 & 0 & {0.5} & 1 & 1 & 1 & 1 \\ 1 & 0 & 0 & 0 & {0.5} & 0 & 0 & 1 \\ 1 & 0 & 0 & 0 & 1 & {0.5} & 0 & 1 \\ 1 & 0 & 0 & 0 & 1 & 1 & {0.5} & 1 \\ {0.3025} & 0 & 0 & 0 & 0 & 0 & 0 & {0.5} \\ \end{array} } \right\}$$

According to the formula (11), the order vector of probability p is.

$${\text{h}}\, = \,(0.0{75},\,0.{1875},\,0.{1696},\,0.{1518},\,0.0{982},\,0.{1161},\,0.{1339},\,0.0{679}).$$

The right ordering vector h and the possibility degree in proof P are obtained, and the ordering of interval number $z_{j} (w)$ is

$${\text{Z}}_{{\text{B}}} \left( {\text{w}} \right)\mathop \ge \limits_{1} {\text{Z}}_{{\text{C}}} \left( {\text{w}} \right)\mathop \ge \limits_{1} {\text{Z}}_{{\text{D}}} \left( {\text{w}} \right)\mathop \ge \limits_{1} {\text{Z}}_{{\text{G}}} \left( {\text{w}} \right)\mathop \ge \limits_{1} {\text{Z}}_{{\text{F}}} \left( {\text{w}} \right)\mathop \ge \limits_{1} {\text{Z}}_{{\text{E}}} \left( {\text{w}} \right)\mathop \ge \limits_{1} {\text{Z}}_{{\text{A}}} \left( {\text{w}} \right)\mathop \ge \limits_{0.6975} {\text{Z}}_{{\text{H}}} \left( {\text{w}} \right).$$

It shows that Yingkou Port ranks the highest in the competitiveness of corn container multimodal transport in Northeast China among Liaoning ports, followed by Jinzhou port, Beiliang port, Panjin Port, Huludao port and Dandong port, and the advantage probability reaches 100%. Dalian port is superior to Suizhong port by nearly 70%, ranking seventh and Suizhong port eighth. In the second level indicators of multimodal transport port hub competitiveness, the weight of business capacity, transport capacity resources, infrastructure and service quality is average, which is 29%, 26%, 24% and 21% respectively. Among the three-level evaluation indicators, the weight of the price of the multimodal transportation of public, railway and hot water is the largest, and the weight of the strategic planning strength of the enterprise is the smallest. Due to national policy guidance, enterprise strategic planning of Liaogang group and market demand, Dalian port is inferior in the supply competition of corn container transportation market in Northeast China, while Yingkou Port has obvious advantages in hinterland distance, transportation price, service maturity and awareness.

## Conclusion

The port hub competitiveness evaluation model based on multi-attribute decision-making constructed in this paper effectively solves the problem of comprehensive evaluation of port hub software and hardware strength in multimodal transport network, and provides a new perspective and method for all-round evaluation of important hubs in multimodal transport complex network. Based on the constructed port hub competitiveness evaluation model, this paper makes an empirical analysis on the distribution of corn container supply in Northeast China. The empirical results have important guiding significance for the competitive and cooperative development of coastal ports in Northeast China. At the same time, Yingkou Port has shown obvious advantages in the supply competition of corn containers in China's northeast coastal ports by virtue of efficient storage system, high-quality transportation services and strong guidance policies, which provides reference and suggestions for port enterprises in enterprise strategic planning and core competitiveness cultivation.

The research results show that the key influencing factors of the competitiveness of the multimodal transport port hub have changed from the traditional infrastructure factors to the flexible influencing factors such as the quality of transport services, the integration ability of transport capacity and resources, and the efficiency of transport business. The port enterprises are in the critical period of industrial transformation and upgrading; Strengthening the integration among industries, building an industrial ecological alliance, and building a multi-mode transport integrated service chain with the port as the core will play an obvious role in promoting the formation of the core competitiveness of the port hub. According to the research results, the following suggestions are put forward. Firstly, based on the strategic goal of China's port sustainable development and combined with the advantageous resources of port enterprises in the region, the government management department should guide port enterprises to adopt the enterprise development model of differentiated development and win–win cooperation, so as to build an ecological environment conducive to the long-term development of port industry. Second, multimodal transport service providers should fully analyze the key factors affecting their competitiveness, and strengthen the core competitiveness of enterprises by optimizing business processes and improving service quality. Third, when choosing the transport service scheme, multimodal transport service users should comprehensively consider the multidimensional factors such as time, cost and safety, and choose the best scheme among the limited resources and limited services.

The shortcomings of this paper and the future research direction. This paper does not make an in-depth analysis of the corn industry chain. The future research will integrate the production and marketing status of the corn industry chain, production and processing layout structure and other factors into the research of port competitiveness to improve the accuracy of the evaluation model. In addition, this paper studies the port competitiveness in public rail water multimodal transport, and the future research can be extended to the competitiveness of railway stations, inland ports and the overall network of multimodal transport.

## Data Availability

The datasets used and/or analyzed during the current study available from the corresponding author on reasonable request.
